# Low carbohydrate intake correlates with trends of insulin resistance and metabolic acidosis in healthy lean individuals

**DOI:** 10.3389/fpubh.2023.1115333

**Published:** 2023-03-16

**Authors:** Fatema Al-Reshed, Sardar Sindhu, Ashraf Al Madhoun, Fatemah Bahman, Halemah AlSaeed, Nadeem Akhter, Md. Zubbair Malik, Fawaz Alzaid, Fahd Al-Mulla, Rasheed Ahmad

**Affiliations:** ^1^Immunology and Microbiology Department, Dasman Diabetes Institute, Kuwait City, Kuwait; ^2^Animal and Imaging Core Facility, Dasman Diabetes Institute, Kuwait City, Kuwait; ^3^Genetics and Bioinformatics, Dasman Diabetes Institute, Dasman, Kuwait; ^4^Institute Necker Enfants Malades (INEM), French Institute of Health and Medical Research (INSERM), Immunity and Metabolism of Diabetes (IMMEDIAB), Université de Paris Cité, Paris, France

**Keywords:** HOMA-IR, C-peptide, low carbohydrate, insulin resistance, anion gap, inflammation

## Abstract

**Introduction:**

Both obesity and a poor diet are considered major risk factors for triggering insulin resistance syndrome (IRS) and the development of type 2 diabetes mellitus (T2DM). Owing to the impact of low-carbohydrate diets, such as the keto diet and the Atkins diet, on weight loss in individuals with obesity, these diets have become an effective strategy for a healthy lifestyle. However, the impact of the ketogenic diet on IRS in healthy individuals of a normal weight has been less well researched. This study presents a cross-sectional observational study that aimed to investigate the effect of low carbohydrate intake in healthy individuals of a normal weight with regard to glucose homeostasis, inflammatory, and metabolic parameters.

**Methods:**

The study included 120 participants who were healthy, had a normal weight (BMI 25 kg/m^2^), and had no history of a major medical condition. Self-reported dietary intake and objective physical activity measured by accelerometry were tracked for 7 days. The participants were divided into three groups according to their dietary intake of carbohydrates: the low-carbohydrate (LC) group (those consuming <45% of their daily energy intake from carbohydrates), the recommended range of carbohydrate (RC) group (those consuming 45–65% of their daily energy intake from carbohydrates), and the high-carbohydrate (HC) group (those consuming more than 65% of their daily energy intake from carbohydrates). Blood samples were collected for the analysis of metabolic markers. HOMA of insulin resistance (HOMA-IR) and HOMA of β-cell function (HOMA-β), as well as C-peptide levels, were used for the evaluation of glucose homeostasis.

**Results:**

Low carbohydrate intake (<45% of total energy) was found to significantly correlate with dysregulated glucose homeostasis as measured by elevations in HOMA-IR, HOMA-β% assessment, and C-peptide levels. Low carbohydrate intake was also found to be coupled with lower serum bicarbonate and serum albumin levels, with an increased anion gap indicating metabolic acidosis. The elevation in C-peptide under low carbohydrate intake was found to be positively correlated with the secretion of IRS-related inflammatory markers, including FGF2, IP-10, IL-6, IL-17A, and MDC, but negatively correlated with IL-3.

**Discussion:**

Overall, the findings of the study showed that, for the first time, low-carbohydrate intake in healthy individuals of a normal weight might lead to dysfunctional glucose homeostasis, increased metabolic acidosis, and the possibility of triggering inflammation by C-peptide elevation in plasma.

## 1. Introduction

Insulin resistance syndrome (IRS) is a modern-day epidemic. With the increase in research endeavors and on the focus on IRS, it has become evident that IRS can drive the disease pathogenesis of several clinical syndromes, such as T2DM and cardiovascular diseases (1, 2). Interestingly, while IRS is often regarded as the primary underlying mechanism for T2DM, several reports from sub-Saharan Africa and South Asian populations indicate that pancreatic beta-cell secretory dysfunction is the driving factor of the lean T2DM phenotype (3, 4). The current recommended dietary guidelines for treating obesity and obesity-related complications revolve around reducing daily energy intake, improving portion control, and improving the quality of the diet to achieve a calorie deficit status (5). However, over the past decade, further research has unraveled the benefits of redirecting the weight loss strategy toward readjusting levels of macronutrients, such as consuming fewer carbohydrates and a larger quantity of proteins in daily meals (5–7). The three macronutrients found in food include carbohydrates (4 kcal/g), proteins (4 kcal/g), and fat (9 kcal/g). A daily intake of <10% or 20–50 g of carbohydrates is considered a very low carbohydrate intake, <26% or <130 g is considered a low carbohydrate intake, 26–44% is considered a moderate carbohydrate intake, and ≥45% is regarded as a high carbohydrate intake (8). There are more than a dozen types of low-carbohydrate diets, of which the ketogenic or keto, Atkins, and paleo diets are relatively more widely known. Keto diets are characterized by reduced carbohydrate content (<50 g per day) and relatively increased fat and protein content. Keto diets are further categorized as follows: (i) standard keto diet (SKD), which contains very low carbohydrate (10%), moderate protein (20%), and high fat (70%) content; (ii) cyclical keto diet (CKD), which involves periods of high-carbohydrate diet in between keto diets, e.g., 5 keto days followed by two high-carbohydrate days as a dietary cycle; (iii) targeted keto diet (TKD), which allows for adding additional carbohydrates around periods of intensive physical workout; and (iv) high-protein keto diet (HPKD), which has a relatively high-protein content (35%) with a low carbohydrate content (5%) but still a high fat (60%) content (9).

With carbohydrates being the macronutrient with the greatest impact on postprandial blood glucose response, low-carbohydrate diets, such as the Atkins and keto diets, have become an effective strategy for weight loss (10, 11). We assumed that, during low carbohydrate intake, the body undergoes ketogenesis, a process that switches the utilization of glucose from the carbohydrate as an energy source to the use of ketone bodies in the mitochondria for ATP synthesis, shifting the metabolic process to the ketosis-favoring pathways (12). This metabolic shift following low-carbohydrate dietary interventions is the cornerstone of weight loss mechanisms. Nevertheless, the use of a low-carbohydrate diet in healthy individuals of a normal weight and in children has been associated with unwanted diet-induced ketoacidosis (13–15). The influence of a low-carbohydrate diet and the activation of the ketogenesis pathway in individuals with obesity, especially those with metabolic complications, such as T2DM, have been proven to be quite effective in reducing body weight (11); however, the significance and adaptation of this lifestyle in individuals of a normal weight in the absence of a family history of diabetes or in those with no history of a major ailment remain unclear. Therefore, the present study aimed to investigate the effect of low carbohydrate intake in healthy, normal weight individuals with regard to glucose homeostasis and inflammatory and metabolic parameters. Herein, we identified that low carbohydrate intake in healthy individuals of a normal weight correlates with dysfunctional glucose homeostasis, increased metabolic acidosis, and the risk of inflammation suggested to be triggered by the elevation in plasma C-peptide levels.

## 2. Materials and methods

### 2.1. Anthropometric, clinical, and dietary characteristics of the study participants

This is a cross-sectional observational study that involved healthy men and women of a normal weight aged 21–65 years. Data were collected between January 2016 and December 2019 and were processed at the Dasman Diabetes Institute, Kuwait. The sample size was determined using the ClinCalc tool software (www.clincalc.com). The incident rate of T2DM onset in individuals of a normal weight is estimated to be 7.7–21% worldwide. Considering 21% as the guiding reference, we achieved a statistical power (1-β) of 95% and a level of significance (α) of 0.05, which yielded the minimum sample size required for this study as 52 individuals. Taking into account a fair margin for possible dropouts, we thus aimed to recruit 100 participants. A total of 138 adult (>18 years) Kuwaiti individuals were reached out to randomly by word of mouth, through flyers, or through social media contacts and were invited to participate in the study. Out of these 138 participants, 120 of them (57 men and 63 women) with a mean age of 31.9 ± 5.7 years and BMI of ≤25 kg/m^2^, were found to be eligible for the final analysis. The study was conducted in accordance with the Helsinki Declaration and the institutional review board ethics of the Kuwait Ministry of Health (MOH) Ethics Board (2017/542) (16). Each participant was required to complete a full-length health screening questionnaire that tracked their past and current health status and history. The health screening questionnaire also asked participants about the health of their immediate family members and inquired about any family-related disease problems. The exclusion criteria were as follows: patients who were physically diagnosed with diabetes, hypertension (>160/90 mmHg), and anti-hypertensive therapy, those with a previous history of established coronary heart disease, e.g., myocardial infarction, coronary artery bypass graft surgery, coronary angioplasty, or a family history of diabetes or early cardiac death (<40 years), those with a history of cancer within the past 2 years, those diagnosed with depression, and those under medications that could influence body weight due to effects on the lipid or carbohydrate metabolism, as well as those who were pregnant or lactating women. A flow chart of participant recruitment is summarized in Supplementary Figure 1A. None of the participants had physical disabilities that would prevent or severely limit physical mobility or physical activity. The characteristics of male and female participants are summarized below in Supplementary Table 1. The presented study follows the Strengthening the Reporting of Observational Studies in Epidemiology (STROBE) recommendations (17).

### 2.2. Physical evaluations

In the physical activity laboratory, a standard protocol was used to carry out all anthropometric assessments for all participants wearing tight-fitting clothes and using the same equipment throughout the study. Measurements were made to the nearest 0.1 unit. Height (cm) was taken by instructing the volunteer to stand with their feet together and back and heels against the upright bar of the height scale. The volunteer was asked to position their head upright against the backboard. The volunteer was requested to take a deep breath as the investigator applied gentle, upward pressure under the angle of the mandible. Other investigators slid down the horizontal bar attached to the scale so that it rested snugly on the examinee's head, and measurements were taken. Body weight (kg) was measured using a beam balance, and BMI was calculated as follows: BMI = weight (kg)/height (m^2^). Waist and hip circumferences (cm) were measured in duplicate using non-elastic tape. Waist circumference was measured at the minimum circumference horizontally between the iliac crest and the rib cage, while hip circumference was measured at the maximum protuberance of the buttocks, and the waist-to-hip ratio was calculated. The same investigator performed these measurements for all volunteers on every occasion. Whole body composition, including body fat percentage, soft lean mass, and total body water, were measured using an IOI 353 Body Composition Analyzer (Jawon Medical, South Korea).

### 2.3. Physical activity measurements

All participants in this study were given an electronic accelerometer (ActiGraph GT3X; ActiGraph LLC, Pensacola, FL, USA) to measure daily physical activity (PA) levels. Subjects were advised to maintain their normal daily habitual PA levels during the study period. The accelerometers were attached to an elasticized belt and worn on the right hip for 7 consecutive days (except when bathing and during water activity). The accelerometer provided PA measurements that included activity counts, vector magnitude, energy expenditure, step counts, PA intensity levels, and metabolic equivalents of tasks (METs). A 1-min epoch was used in this study with activity counts assessed at 1-min intervals to ensure that the data quality for the participants included at least 4 days in which the accelerometer was worn for at least 60% of the time of the day. A non-wear time was taken as any block of time ≥60 min wherein the activity count was equal to zero (18). Individuals that did not meet those criteria were excluded from the study, and their collected data were removed from the data pool.

Freedson's cutoffs (14) were used to differentiate between PA intensity levels, including light-intensity activity (100–1,951 counts/min), moderate-intensity activity (1,952–5,724 counts/min), and high-intensity activity (>5,725 counts/min). All counts ≤99 counts/min were considered sedentary. The data were also expressed as the mean intensity for each activity during the monitoring time (total accelerometer counts per total monitoring time).

### 2.4. Measurement of metabolic and inflammatory markers

Volunteers were asked for a second visit after an overnight fast of at least 10 h. Blood pressure and heart rate were measured for each participant using a semiautomatic Omron portable monitor. In brief, the cuff was placed on the upper arm to ensure uniform compression of the brachial artery, and three consecutive readings were collected (19). Blood samples were collected in 10 mL Ethylenediaminetetraacetic acid (EDTA) tubes (BD Vacutainer system, Plymouth, UK). Plasma was separated and frozen immediately at−80°C for further analysis. Total blood glucose, fasting plasma insulin, cholesterol, HDL-cholesterol, and triglycerides were determined by biochemical analysis using a single assay upon the completion of the sampling (refer to Supplementary Table 2 for information regarding normal ranges). Quality control sera were used to monitor the accuracy and precision of the assays.

Quantitative insulin sensitivity indices, HOMA-IR and HOMA-β, were calculated as follows:


$$\begin{array}{l} \text{HOMA}-\text{IR }=\text{ fasting insulin }(\text{μU}/\text{L})\;\times \text{ fasting glucose}\\ \;(mmol/L)/22.5\\ \text{HOMA}-\text{beta}-\text{cell function}(\text{HOMA}-\text{β})\text{\%}\\ \;=360\times \text{ fasting insulin }(\text{μU}/\text{mL})\\ \;/(\text{fasting glucose }(\text{mg}/\text{dL})\;-\;63) \end{array}$$

The anion gap was calculated according to the following equation:


$$\begin{array}{l} \text{ Anion gap = serum sodium }(\text{mmol/l})\\ \;-[\text{serum chloride }(\text{mmol/l})\\ \text{ + serum bicarbonate }(\text{mmol/l})]\\ \text{Albumin corrected anion gap = anion gap + }[\text{2}.\text{5 }\times \;(\text{4}\\ \;-albumin,\;g/dL)] \end{array}$$

### 2.5. Dietary monitoring and analysis

All participants were given food diaries and were instructed to weigh and record their daily intake of food and drinks on electronic scales for the length of the study (7 days) (Salter Housewares, Kent, United Kingdom). A visual demonstration of how to use scales and diaries was given to each individual prior to the start of the study. All individuals were advised to maintain their normal dietary intake. Diaries were completed prior to the second visit. Food diary data were analyzed using CompEat pro (Nutrition systems, Banbury, United Kingdom), and an average of the daily nutrient intake was calculated. According to the international health guidelines established by the Food and Nutrition Board of the National Academies of Sciences, Engineering, and Medicine for the Recommended Dietary Allowance (RDA) for carbohydrates, the recommended daily energy intake from carbohydrates is set between 45 and 65% of daily calorie intake since this amount has been linked to a lower risk of chronic illnesses (15, 20). Based on these criteria, study participants were divided into three groups as follows: the low-carbohydrate (LC) group (those consuming <45% of daily energy intake from carbohydrates), the recommended range of carbohydrate (RC) group (those consuming 45–65% of daily energy intake from carbohydrates), and the high-carbohydrate (HC) group (those consuming higher than 65% of daily energy intake from carbohydrates).

### 2.6. Enzyme-linked immunosorbent assay

Commercially available ELISA kits were used for the detection of plasma levels of fasting insulin and C-peptide (Mercodia, Uppsala, Sweden), following instructions from the manufacturers.

### 2.7. Determination of plasma cytokines/chemokines

A total of 41 cytokines and chemokines were measured using the MILLIPLEX MAP Human Cytokine/Chemokine panel with Magnetic Bead Panel-Premixed 41 Plex-Immunology Multiplex Assay (Milliplex map kit, HCYTMAG-60 K-PX41; Millipore, USA), following the manufacturer's instructions. Data from the reactions were acquired by Luminex using a MILLIPLEX analyzer, while a digital processor managed the data output. MILLIPLEX Analyst software was used to determine the mean fluorescence intensity (MFI) and analyte concentration (pmol/mL).

### 2.8. Statistical analysis

Data were analyzed using SPSS version 25 (SPSS, Inc., Chicago, IL) and GraphPad Prism 7.01 (version 6.05; San Diego, CA, USA) and expressed as the mean ± standard deviation (SD). The data were tested for normality using the Shapiro–Wilk normality test. For comparing the means between two groups, two-tailed *t*-tests and Wilcoxon–Mann–Whitney U tests were used to assess the differences between means of parametric and non-parametric data, respectively. For comparing the means between the three groups, a one-way ANOVA and exact Kruskal–Wallis tests were used when comparing the differences between the means of parametric and non-parametric data, respectively. Multiple linear regression analysis was conducted to examine the correlation between the calculated anion gap and blood electrolyte levels that were found to be associated with LC intake. Exact chi-squared tests of independence were performed to evaluate differences in immune–metabolic parameters. The correlation between energy intake from carbohydrates and immune–metabolic parameters was evaluated with Spearman's correlation coefficients. All *p*-values of ≤0.05 were considered statistically significant.

## 3. Results

### 3.1. Participants' characteristics

A total of 138 people were invited to participate in the ActiGraph track assessments. Following a comprehensive health screening, only 134 individuals were found to meet the inclusion health criteria. Of those 138 individuals, only 120 of them (57 men and 63 women) had sufficient data from the ActiGraph that included at least 4 days in which the accelerometer was worn for at least 60% of the time. The general characteristics of the study participants and dietary and energy intake data are summarized in Supplementary Table 3. Based on the WHO chart for age and sex, all participants were within the normal range of BMI, with an average BMI of 22.7 ± 2.4 kg/m^2^. In our study, 52.5% of participants were women, and the mean age of all participants was 32.2 ± 5.7. The mean systolic and diastolic blood pressure measurements were normal (109.9 ± 11.3 and 67.15 ± 9.8, respectively), and the average heart rate per min (HR) was 71 ± 10. The study participants had normal levels of serum triglycerides (TG) (0.87 ± 0.38 mmol/L), total cholesterol (4.6 ± 0.8 mmol/L), and HDL-C (1.49 ± 0.34 mmol/l). All participants also showed normal glucose homeostasis, with an average fasting glucose of 4.9 ± 0.64 mmol/L and a serum insulin level of 3.7 ± 2.11 U/ml. All individuals were within the normal range of HOMA-β (%) (74.8 ± 58.9) and HOMA-IR (<2.5) indices. The participants also had normal fasting blood C-peptide levels (1.4 ± 0.37 ng/mL). The average total calorie intake per day for all participants was 2143.8 ± 571.9 kcal, with most energy consumed from carbohydrates at 49.5 ± 12.5% of daily energy.

To explore the contribution of the level of carbohydrate energy intake on the general health of the participants, we decided to pool both the male and female data. Based on the international health guidelines of daily RDA, study participants were divided into three groups as follows: the low-carbohydrate (LC) group (those consuming <45% of daily energy intake from carbohydrates), the recommended range of the carbohydrate (RC) group (those consuming 45–65% of daily energy intake from carbohydrate); and the high-carbohydrate (HC) group (those consuming higher than 65% of daily energy intake from carbohydrate). Group characteristics are summarized in Table 1, which shows significant cross-group differences with regard to lean weight (LC vs. RC and HC), HOMA-beta-cell function (HOMA-β%) (LC vs. RC), fasting glucose, insulin, C-peptide, and the homeostasis model assessment of insulin resistance (HOMA-IR) index (LC vs. RC and RC vs. HC). Concurrently, no significant differences were found regarding anthropometric characteristics, lipid profile, and total calorie intake per day.

**Table 1:** Differences between groups based on daily calorie intake (%) from carbohydrates.

**Physical characteristics of subjects**	**<45% (LC)**		**45–65% (RC)**		**>65% (HC)**		***p*-value**
	**(*****n*** = **38) 20 M/18 F**	**Median-IQR**	**(*****n*** = **62) 30 M/32 F**	**Median-IQR**	**(*****n*** = **20) 7 M/ 13 F**	**Median-IQR**	
Age (years)	32 ± 4		31 ± 4		33 ± 6		0.551
Weight (kg)	64.1 ± 10.1		66.1 ± 12.8		63.0 ± 13.7		0.538
Height (cm)	167.8 ± 11.4		170.1 ± 11.6		163.4 ± 12.5		0.090
BMI (kg/m^2^)	22.7 ± 2.9	22.9–43	22.9 ± 2.4	22.6–2.9	23.2 ± 2.6	22.4–3.2	0.796
Waist circumference (cm)	75.9 ± 9.1		80.5 ± 7.8		79.5 ± 13.7		0.079
Hip circumference (cm)	105.1 ± 30.7	39.2–4.6	100.5 ± 10.1	39.7–5.2	100.5 ± 9.6	40–3.2	0.483
Fat weight (kg)	20.3 ± 11.2		23.4 ± 12.1		27.8 ± 11.6		0.074
Lean weight (kg)	49.5 ± 11.6	51–23.1	43.4 ± 11.4	45.2–10.5	42.2 ± 9.3	43.1–13	**0.0075**
Fat %	21.3 ± 10.3		25.9± 10.0		26.8 ± 7.3		0.092
Total calorie intake (Kcal)	2104.7 ± 581		2124 ± 588		2277 ± 504		0.514
BP/systolic (mmHg)	110.6 ± 11.7	110–14	109.3 ± 11.1	108.5–14	110.9 ± 11.6	110–10.5	0.800
BP/diastolic (mmHg)	67.7 ± 10.4	70–18.2	66.5 ± 10.2	68–16.2	67.8 ± 7.1	67–4	0.810
Heart rate	70 ± 11	71–20.5	70 ± 10	73–15	74 ± 9	76–12.1	0.381
Fasting glucose (mmol/l)	5.2 ± 0.7	5.1–0.86	4.7 ± 0.5	4.7–0.60	5.5 ± 0.5	5.2–0.85	**0.0008**
Triglycerides (mmol/l)	0.8 ± 0.3	0.9–0.43	0.8 ± 0.4	0.7–0.48	0.8 ± 0.3	0.9–0.47	0.602
Total cholesterol (mmol/l)	4.5 ± 0.7	4.5–0.86	4.6 ± 0.7	4.5–0.77	4.9 ± 1.0	4.83–0.96	0.469
HDL cholesterol (mmol/l)	1.5 ± 0.32	1.3–0.53	1.45 ± 0.37	1.3–0.63	1.6 ± 0.3	1.5–0.54	0.064
Insulin Con. (mU/l)	4.5 ± 1.4	4.4–2.0	3.2 ± 2.3	2.7–4.1	4.6 ± 2.2	5.0–3.0	**0.001**
HOMA-IR	1.0 ± 0.37	1.0–0.42	0.70 ± 0.47	0.6–0.7	1.0± 0.55	1.0–0.57	**0.013**
HOMA-β	56.3 ± 30.3	52.2–42.9	80.5 ± 59.4	45.7–68.8	58.9 ± 37.0	51.8–56.8	**0.016**
C-Peptide (pg/ml)	1.5 ± 0.36	1.4–0.64	1.3 ± 0.34	1.3–0.37	1.56 ± 0.36	1.6–0.62	**0.019**

Results are presented as mean ± SD. BMI, body mass index; BP, blood pressure; HDL, high-density lipoprotein; HOMA-IR, homeostatic model assessment for insulin resistance. The bold values indicate significant values with *p* < 0.05 for easier identification.

**Figure 1:**
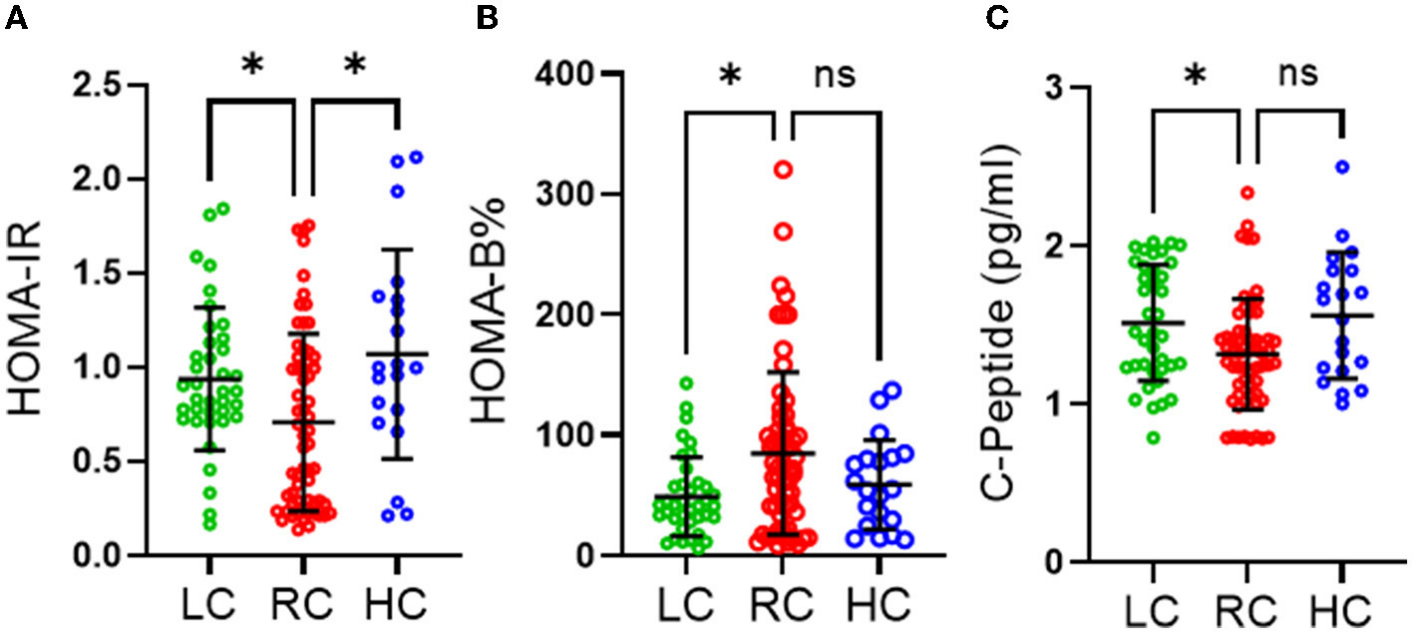
Effects of different levels of carbohydrate intake on glucose homeostasis. **(A)** HOMA-IR homeostasis model assessment of insulin resistance, **(B)** HOMA-beta homeostasis model assessment of β-cell function **(C)** C-peptide section. Data are expressed as mean ± SD. Data analyzed by exact Kruskal-Wallis test. ns, non-significant, *P ≤ 0.05. Low carbohydrate (LC) (those consuming <45% of daily energy percentage) represented in green, recommended range of carbohydrate (RC) (those consuming 45–65% of daily energy percentage) represented in red and high carbohydrate (HC) (those consuming higher than 65% of daily percentage) represented in blue.

We also found no significant differences in the level of objectively measured physical activity, as indicated in Table 2. However, individuals consuming RC were found to have significantly lower HOMA-IR than those consuming LC (*p* ≤ 0.05) and HC (*p* ≤ 0.05) and significantly higher HOMA-β (%) than those consuming LC (*p* ≤ 0.05) (Figures 1A, B). It was also observed that participants consuming RC had significantly lower C-peptides in their serum than in the LC and HC groups. However, only LC was found to be significantly higher than RC (*p* < 0.05) (Figure 1C).

**Table 2:** Comparison of physical activity levels across groups.

**Physical activity level**	**>45% (LC)**		**45–65% (RC)**		**<65% (HC)**		***p*-value**
	**(*****n*** = **38) 20 M/18 F**	**Median-IQR**	**(*****n*** = **62) 30 M/32 F**	**Median-IQR**	**(*****n*** = **20) 7 M/ 13 F**	**Median-IQR**	
Overall activity (%)	29.2 ± 6.2	70–15.5	30.3 ± 7.5	69.1–7.3	32.4 ± 6.3	66.9–11.4	0.244
Light intensity (%)	22.5 ± 4.5	22.5–6.0	23.9 ± 5.2	23.1–6.4	25.4 ± 5.8	25.6–9.8	0.107
Moderate intensity (%)	5.6 ± 2.3	5.3–2.8	5.9 ± 2.4	5.3–3.0	5.7 ± 1.2	5.48–1.9	0.807
Vigorous intensity (%)	1.0 ± 0.9	0.7–0.96	0.8 ± 0.7	0.6–0.5	1.1 ± 0.6	0.9–1.0	0.372
Average MET rate/day	1.5 ± 0.1	1.5–0.18	1.5 ± 0.2	1.5–0.13	1.5± 0.1	1.6–0.17	0.350
Average step count/day	9,541 ± 3,608	9087–6693.5	1,1781 ± 17,031	9,207–5573.5	10,134 ± 2,174	10,867–4,009	0.666

Results are presented as mean ± SD, MET, metabolic equivalent of task.

**Figure 2:**
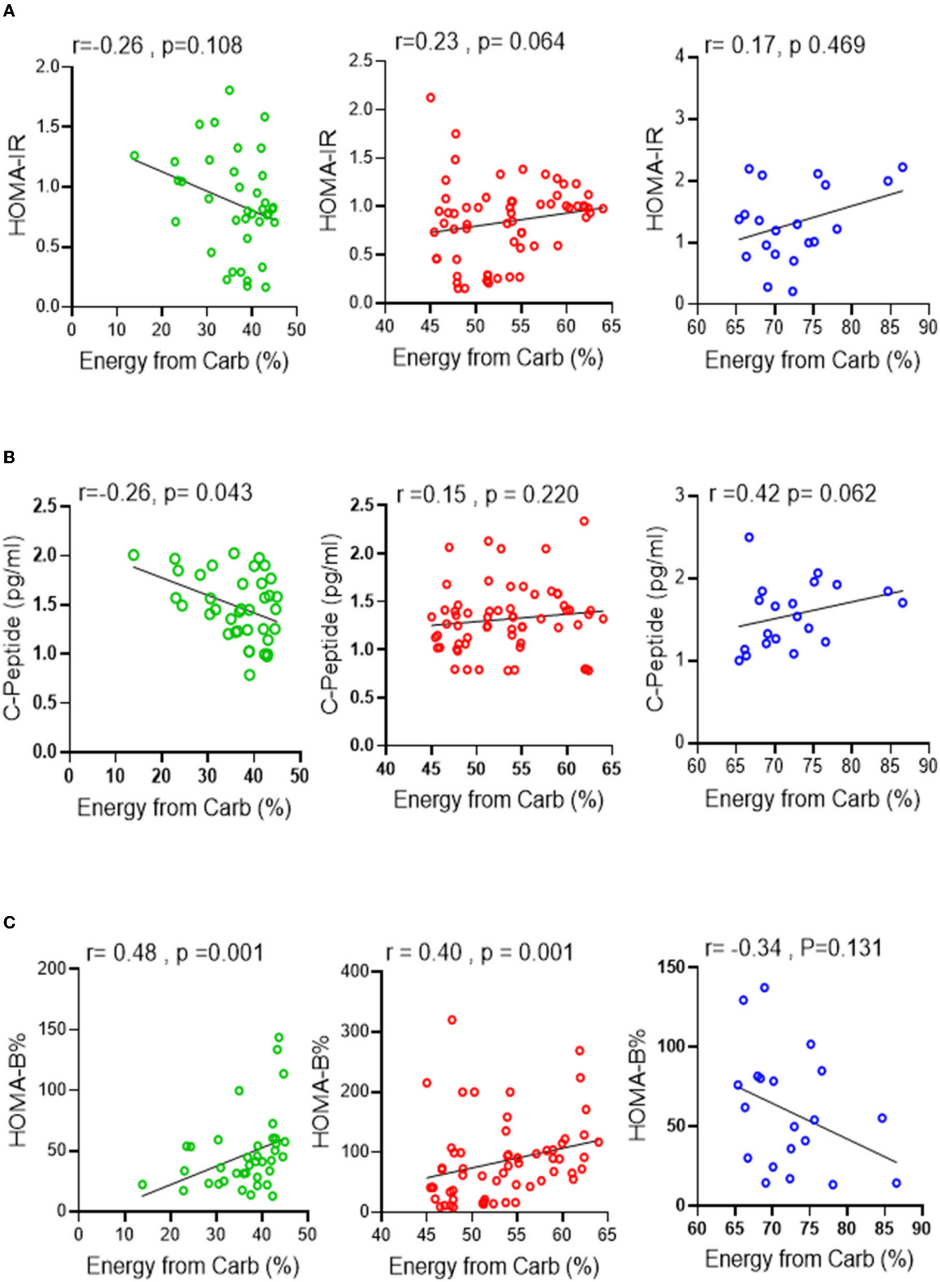
Association between carbohydrate intake levels and glucose homeostasis. Spearman correlation test was conducted to investigate the association between **(A)** HOMA-IR, **(B)** C-peptide secretion and **(C)** HOMA-β% and the level of carbohydrate intake % of energy in all three groups. All data are expressed as mean ± SD. *P* ≤ 0.05 was considered statistically significant. Low Carbohydrate intake (<45% energy from carb; green), Recommended range of carbohydrate intake (45–65% energy from carb; red), High carbohydrate intake (>65% energy from carb; blue).

To further investigate these findings, a Spearman correlation test was performed to determine the correlation between carbohydrate energy % and surrogate markers of insulin resistance, β-cell function, and insulin secretion [HOMA-IR, HOMA-β (%), and c-peptide levels, respectively]. No significant correlation was found between HOMA-IR and carbohydrate energy % across all groups (Figure 2A). However, unlike the RC and HC groups, a clear trend of negative HOMA-IR associated with carbohydrate energy % was observed in the LC group. Interestingly, fasting serum C-peptide levels in the LC group had a significant negative correlation (*p* ≤ 0.05) with carbohydrate energy %, while C-peptide levels in the RC and HC groups tended to have a direct correlation with carbohydrate energy % (Figure 2B). In terms of β-cell function assessment, the HOMA-β% index was associated positively with levels of carbohydrate energy % in both LC and RC groups, whereas HOMA-β% tended to have a negative correlation with carbohydrate energy % in the HC group (Figure 2C). On the whole, these data clearly indicate that low carbohydrate intake might be correlated with insulin resistance and that the consumption of 45–65% of energy intake from carbohydrates is important to maintain normal glucose hemostasis in individuals of a normal weight.

### 3.2. Association of the percentage of energy intake from dietary carbohydrate with the serum anion gap marker for metabolic acidosis

**Figure 3:**
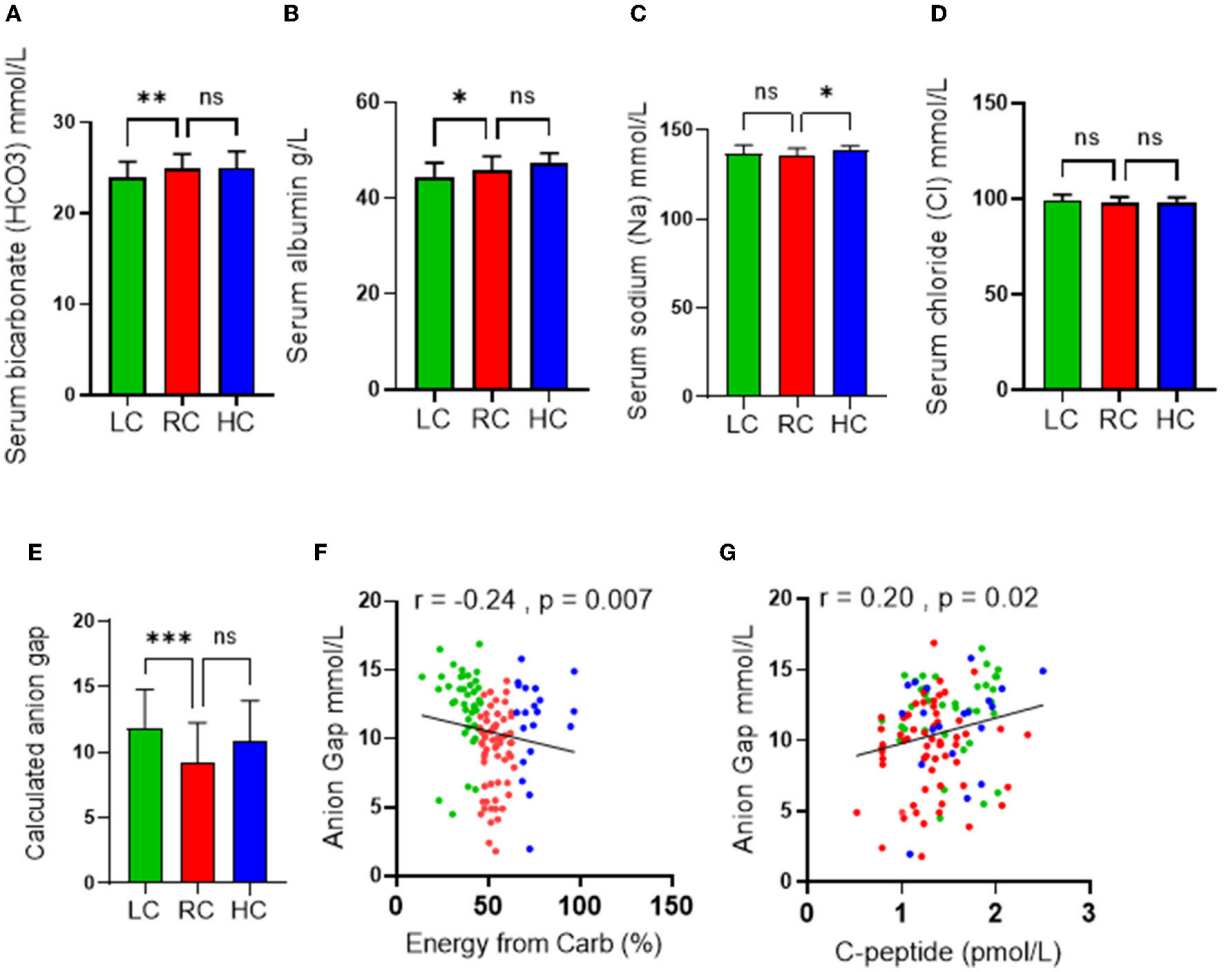
Effects of the levels of carbohydrate intake on metabolic acidosis. Serum levels of **(A)** Bicarbonate; **(B)** Albumin; **(C)** Chloride; and **(D)** Sodium are shown in individuals with low-carbohydrate (LC group: <45% of daily energy from carbohydrate), recommended range of carbohydrate (RC group: 45–65% of daily energy from carbohydrate) and high-carbohydrate (HC group: >65% of daily energy from carbohydrate) intake. **(E)** Calculated anion gap is shown for individuals in LC, RC, and HC dietary groups. **(F)** Association (Spearman r correlation test) between anion gap and carbohydrate intake. **(G)** Association (Spearman r correlation test) between anion gap and C-peptide. Data are expressed as mean ± SD. ns, non-significant; *p ≤ 0.05, **p ≤ 0.01, ***p ≤ 0.005.

Because of the role of ketone bodies in causing acid-base disruption, measuring plasma electrolytes and calculating anion gap became standard clinical practice for the evaluation of metabolic acidosis. Similar to other metabolic blood markers, the mean values of serum bicarbonate (24 ± 1.7 mmol/l), serum albumin (64.3 ± 2.8), serum sodium (Na) (136.5 ± 3.9 mmol/l), serum chloride (Cl) (98.3 ± 2.9), and calculated anion gap (10.5 ± 3.2) were all within ranges considered normal. However, the LC group displayed significantly lower serum bicarbonate and serum albumin levels (Figures 3A, B) than the RC group. The HC group was also found to have significantly upregulated serum sodium levels compared to the RC group only (Figure 3C), while no significance was found in the level of serum chloride across all groups (Figure 3D). Both disturbances of serum bicarbonate and albumin are considered signs of metabolic acidosis. Indeed, the calculated anion gap further reflected a significant upregulation in metabolic acidosis in the LC group compared to the RC group, with trends of upregulation in the HC group being found to not be significant (Figure 3E). Through the use of the Spearmen *r* coefficient, it was observed that the serum anion gap was inversely associated with the percentage of energy intake consumed from carbohydrates (Figure 3F); we also found a negative correlation between the anion gap and C-peptide (Figure 3G). As indicated by multilinear regression analyses, both serum albumin levels as well bicarbonates were found to be associated independently with the calculated anion gap (Table 3). Together these observations indicate an increase in high anion gap metabolic acidosis triggered by imbalanced serum bicarbonate and albumin under the consumption of a low-carbohydrate diet.

**Table 3:** Multiple regression analysis.

	**Blood electrolytes levels**	**Standardized coefficient β**	**95% confidence interval**	***p*-value**
Calculated anion gap	Albumin g/L	0.3984	“0.9181 to 2.496”	**<0.0001**
	*Sodium (Na) mmol/L*	0.2998	“-0.3829 to 0.8046”	0.4832
	Chloride (Cl) mmol/L	0.4092	“-0.1723 to 1.449”	0.1216
	Bicarbonate	0.3984	“1.029 to 3.768”	**0.0007**

The bold values indicate significant values with *p* < 0.05 for easier identification.

### 3.3. Association of the C-peptide levels with circulatory inflammatory markers

**Figure 4:**
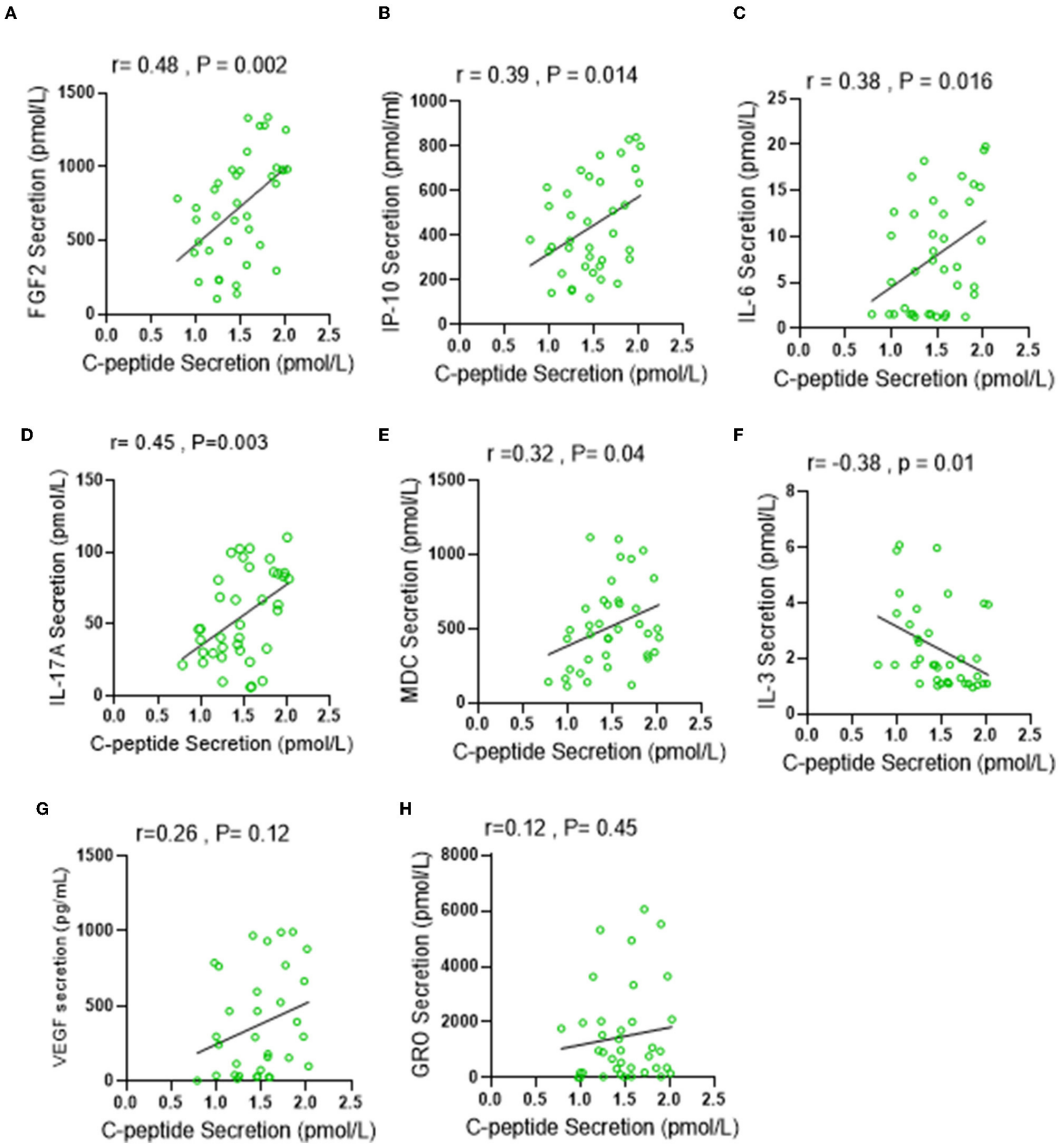
Low carbohydrate (LC) intake is associated with IRS-related inflammatory markers under low carbohydrate intake. Plasma levels of IRS related secretion of 41 cytokine and chemokine were determined by multiplex Assay. **(A–H)** Spearman correlation analysis was conducted to investigate the relationship between significantly elevated cytokines in LC group and C-peptide secretion levels. All data are expressed as mean ± SD. *P* ≤ 0.05 was considered statistically significant.

C-peptide is a biologically active short polypeptide (31 amino acids) that serves as a diagnostic biomarker to distinguish between type 1 and type 2 diabetes and is a strong indicator of insulin biosynthesis and insulin resistance syndrome (IRS) (21–23). Over the past decade, several studies demonstrated a biological effect of plasma circulating C-peptide on activating inflammatory signaling pathways (24, 25). Thus, we questioned the possible association of elevated levels of C-peptide under low carbohydrate intake with insulin resistance-related inflammatory cytokines. A multiplex cytokine assay was conducted to investigate the secretion of these cytokines, known to be involved in several metabolic disorders, such as diabetes and insulin resistance syndrome. Out of the 41 inflammatory mediators investigated, only seven of them (IP-10; *p* = 0.045, VEGF; *p* = 0.049, IL-6; 0.049, IL-17A; *p* = < 0.0001, FGF-2; *p* = 0.025, MDC; *p* = 0.019, and GRO; *p* = 0.035) were found to be significantly elevated in the LC group when compared with the RC group, and only one cytokine (IL-3; *p* = 0.036) was found to be significantly reduced in the LC group when compared with HC group, as depicted in Table 4. The Spearmen correlation analysis further showed that, out of those eight cytokines/bioactive factors, five were found to be positively correlated with C-peptide expression (FGF-2; *r* = 0.52, *p* = 0.001, IP-10; *r* = 0.33, *p* = 0.04, IL-6; *r* = 0.31, *p* = 0.05, IL-17A; *r* = 0.39, *p* = 0.015, and MDC; *r* = 0.36, *p* = 0.025) (Figures 4A–E), one was negatively correlated with C-peptide expression (IL-3; *r* = 0.45, *p* = 0.005) (Figure 4F), and two (VEGF and GRO) had no correlation with C-peptide levels (Figures 4G, H). Together, the presented data suggest that, under the condition of low dietary carbohydrate intake, a correlation is found between the plasma C-peptide levels and IRS-related cytokine/mediator expression, supporting the active role of C-peptide as a bioactive molecule and its significance as an IRS biomarker.

**Table 4:** Group-based comparison of plasma inflammatory markers.

**Plasma inflammatory markers**	**<45% (LC)**		**45–65% (RC)**		**>65% (HC)**		***p*-value**
	**(*****n*** = **38)**		**(*****n*** = **62)**		**(*****n*** = **20)**		
	**20 M/18 F**	**Median-IQR**	**30 M/32 F**	**Median-IQR**	**7 M/ 13 F**	**Median-IQR**	
EGF (pmol/L)	211.5 ± 229.7	158.5–330.3	139.7 ± 156.0	146.5–231	95.0 ± 102.9	75–173.1	0.198
Eotoxin (pmol/L)	112.3 ± 110.5	74.2–215.8	101.0 ± 105.2	51.8–204.5	68.6 ± 69.1	63.8–142	0.512
FGF-2 (pmol/L)	723.2 ± 350.8	492–686.6	494.0 ± 425.4	297–829.0	457.3 ± 365.8	464.5–767.5	**0.025** ^**Ψ**^
Fit-3L (pmol/L)	13.9 ± 24.7	1.4–4.23	8.3 ± 19.7	1.4–1.0	13.6 ± 38.5	1.4–1.0	0.716
Fractalkine (pmol/L)	244.4 ± 695.7	0.3–139.9	144.0 ± 238.6	0.3–137.6	206.4 ± 624.6	0.3–72.2	0.779
G-CSF (pmol/L)	126.2 ± 205.6	35.8–295.1	239.6 ± 210.2	32.7–359.4	71.7 ± 118.6	13.5–118	0.474
GM-CSF (pmol/L)	13.9 ± 29.8	8.3–8.87	17.9 ± 24.8	9.4–17.2	25.9 ± 62.6	6.1–3.7	0.678
GRO (pmol/L)	1282.5 ± 1687.8	51.2–1390.3	1051.4 ± 2279.2	64.9–1324	1530.1 ± 1930.6	654.3–1581.8	**0.035** ^**Ψ**^
IFN-α2 (pmol/L)	112.6 ± 119.4	72.1–215.1	143.3 ± 173.7	102.1–170.9	169.7 ± 179.6	151–255	0.637
IFN-γ (pmol/L)	1196.5 ± 2417.1	642–1069.5	1054.3 ± 1857.7	599–1569.7	1059.3 ± 1700.3	441–1331.2	0.970
IL-10 (pmol/L)	29.3 ± 56.4	1.4–38.5	41.5 ± 56.3	1.8–88.4	28.9 ± 42.8	2.5–63.6	0.680
IL-13 (pmol/L)	36.0 ± 98.2	8.1–15.6	18.8 ± 65.8	1.6–3.5	37.3 ± 44.3	1.6–0.1	0.586
IL-12P40 (pmol/L)	5.2 ± 6.99	1.6–97.3	7.22 ± 13.5	1.6–4.3	21.2 ± 66.0	1.6–2.8	0.351
IL-12P70 (pmol/L)	105.1 ± 241.0	1.6–97.3	65.6 ± 170.9	1.6–26.4	37.7 ± 90.7	1.6–20.8	0.603
IL-15 (pmol/L)	4.77 ± 10.9	1.6–3.1	3.7 ± 4.6	1.6–5.3	4.1 ± 4.3	1.6–7	0.769
IL-17A (pmol/L)	55.6 ± 30.6	37.7–35.7	27.4 ± 21.2	28.4–25.4	48.5 ± 29.2	41.9–64.9	**<0.0001** ^**ΨΔ**^
IL-1RA (pmol/L)	134.1 ± 228.5	73.3–166.6	118.2 ± 170.5	50.6–204.6	101.9 ± 114.0	92.5–148.1	0.893
IL-1α (pmol/L)	50.1 ± 90.4	8.4–50.6	51.8 ± 123.1	9.1–31.1	72.4 ± 107.8	7.6–146.4	0.839
IL-1β (pmol/L)	6.9 ± 6.2	5.0–7.5	7.8 ± 11.6	4.7–7	7.1 ± 8.3	3–8.6	0.902
IL-2 (pmol/L)	2.2 ± 1.0	1.7–0.94	2.9 ± 2.0	1.7–1.6	6.8 ± 6.9	7.2–23.7	0.087
IL-3 (pmol/L)	2.3 ± 1.5	1.7–0.6	9.2 ± 13.5	1.3–0.07	2.9 ± 4.7	1.7–0.6	**0.0367** ^**Ψ**^
IL-4 (pmol/L)	1.2 ± 2.4	1.1–0	2.3 ± 1.9	1.1–1.2	1.1 ± 2.3	1.1–0	0.149
IL-5 (pmol/L)	13.6 ± 29.6	2.43–7.2	7.5 ± 15.9	1.4–2.1	12.9 ± 13.9	4.6–27.2	0.743
IL-6 (pmol/L)	7.8 ± 6.1	6.5–11.4	5.5 ± 5.1	3.2–7.9	6.2 ± 7.3	3.7–8.3	**0.0495** ^**Ψ**^
IL-8 (pmol/L)	24.6 ± 27.2	15.6–31.1	16.8 ± 19.0	9.9–16	15.0 ± 21.0	6.5–32.3	0.169
IL-9 (pmol/L)	240 ± 382.7	14.6–641.0	244.9 ± 342.0	26.5–519.3	155.5 ± 250.1	13.1–263.5	0.721
IP-10 (pmol/L)	441.9 ± 215.6	262–303	375.2 ± 283.5	283–351	413.6 ± 241.4	322–374.7	**0.045** ^**Ψ**^
MCP-1 (pmol/L)	318.7 ± 218.4	269.5–304.7	391.1 ± 231.0	239.5–260	272.6 ± 193.8	266.5–284.5	0.738
MCP-3 (pmol/L)	17.6 ± 20.7	1.9–36.9	26.4 ± 74.3	1.4–12.6	10.2 ± 26.8	1.4–6.3	0.676
MDC (pmol/L)	514.2 ± 285.6	217–778.3	489.5 ± 436.9	612–800	546.1 ± 482.7	358.8–909.6	**0.019** ^**Ψ**^
MIP-1α (pmol/L)	25.8 ± 24.2	15.2–36.7	22.6 ± 20.3	16.4–32.6	25.0 ± 21.7	19.9–27.4	0.765
MIP-1β (pmol/L)	43.7 ± 54.5	24.3–29.3	32.2 ± 34.4	20–41.3	40.9 ± 47.7	24.7–49.7	0.538
sCSD40L (pmol/L)	2286.0 ± 3223.5	124–4563.7	2419.0 ± 3347.7	114–3503.7	2559.3 ± 2898.6	1753.8–5167.9	0.975
TGF-α (pmol/L)	44.1 ± 79.9	9.2–686.6	35.3 ± 57.9	8.9–51.1	12.6 ± 20.7	5.8–4	0.368
TNF-α (pmol/L)	71.7 ± 74.7	56.9–59.7	64.8 ± 82.9	41–55.1	69.1 ± 26.4	51.7–45.3	0.901
TNF-β (pmol/L)	13.5 ± 12.1	12.1–17.7	11.1 ± 11.2	8.8–17.4	11.7 ± 11.1	4.8–11.5	0.774

Results are presented as mean ± SD. ^Ψ^LC vs. RC, and ^Δ^RC vs. HC. The bold values indicate significant values with *p* < 0.05 for easier identification.

## 4. Discussion

The combination of many mechanisms, including homoeostatic, environmental, and behavioral, regulates body weight. The hypothalamus is central to homoeostatic control because it integrates information about food intake, energy balance, and body weight. However, an “obesogenic” environment and behavioral patterns influence the amount and kind of food consumed and physical activity. Unfortunately, physiological weight loss adaptations have been found to favor weight recovery (26). These modifications include changes in the circulatory levels of hunger-related hormones, energy homeostasis, nutrition metabolism, and subjective appetite (27). Notably, individuals need to adhere to behaviors that resist physiological adaptations and other variables that favor weight recovery to successfully sustain weight reduction. With the global rise of obesity and T2DM in humans, various dietary strategies that target the restriction of calorie intake have been used not only to promote weight loss but also to prevent and reduce the onset of T2DM (28, 29). Over the past decade, a low-carbohydrate diet has been centered on weight loss in individuals with obesity and those who are overweight, as well as in patients with or at risk of T2DM (30, 31). Even though the impact of a low-carbohydrate diet, especially the ketogenic diet, has been found to be very effective in the rapid induction of weight loss in both individuals with obesity and those who are overweight, the impact of such a diet remains to be well characterized in normal weight or lean counterparts.

In this study, to the best of our knowledge, we investigated, for the first time, the effect of different dietary carbohydrate intake levels on glucose hemostasis, blood electrolyte balance, and T2DM-related inflammatory markers in 120 individuals of a normal weight (BMI ≤ 25 kg/m^2^). The data presented herein show that individuals with low carbohydrate intake, i.e., those consuming ≤45% of their daily calorie intake from carbohydrates, presented with the trends of IRS. We found that, under the condition of low carbohydrate intake, plasma insulin levels and, consequently, the HOMA-IR were both significantly elevated compared to weight-matched counterparts that consumed sufficient levels of carbohydrate for energy (45–56% of daily calorie intake), while the HOMA index representing beta-cell function (HOMA-β%) was found to be decreased under low carbohydrate intake diet. In this regard, we observed increased plasma insulin levels and HOMA-IR values in individuals of a normal weight who had low dietary carbohydrate intake, which may explain why proteolytic and lipolytic responses are enhanced under low dietary carbohydrate intake as part of alternate compensatory mechanisms to generate glucose from amino acids and glycerol (32). Such gluconeogenic responses following a carbohydrate-restricted diet could be helpful for maintaining glycemia in healthy individuals; however, exacerbated glucose production and ketogenesis remain the major concerns involved (33). Carbohydrate restriction to very low levels may also have deleterious effects on intestinal homeostasis and fiber-derived antioxidant phenolic acids compared with a moderate or high carbohydrate intake (34). Furthermore, a relative increase of ketone concentrations under low dietary carbohydrate intake may at first stimulate the pancreas to increase insulin release, which may accumulate metabolic stress over time (35). In addition, carbohydrate restriction induces lipolysis, releases free fatty acids, and increases citric acid cycle flux, all of which are known reasons to promote reactive oxygen species (ROS) production (36) and suppress the function of beta cells (37), which may be explained by the lower HOMA-β% values that we observed in individuals of a normal weight on low dietary carbohydrate intake.

Interestingly, plasma C-peptide levels were also found to be significantly elevated under low carbohydrate intake. A significant correlation was found between glucose homeostasis markers and low dietary carbohydrate intake, further supporting the effect of low-carbohydrate diet intake on glucose homeostasis. Carbohydrate metabolism is a fundamental biochemical process that ensures a constant supply of energy to living cells. With the prolonged consumption of low carbohydrate intake, the liver starts to produce ketone bodies as an alternative source of energy. Ketone bodies travel from the liver to extrahepatic tissues to provide energy to different organs by breaking down fatty acids and ketogenic amino acids (38, 39). In the studied cohort, an elevation in the anion gap was observed in the LC group. Through further multi-regression analysis, it was discovered that this elevation was caused by the reduction in both serum bicarbonate and serum albumin levels. An association was also observed between the level of anion gap and the level of energy from carbohydrate intake. It is irrefutable that any diet based on restrictions and exclusions of certain foods will induce a possible increase in the risk of mineral deficiencies and electrolyte imbalance. In fact, studies have shown that consuming a low-carbohydrate diet while maintaining a high intake of protein can lead to a disturbance in fluid and electrolytes, which can further cause kidney damage (40, 41).

Interestingly, in our cohort, we also highlight a correlation between the anion gap and C-peptide levels. C-peptide is the part of proinsulin that is cleaved from pancreatic beta cells prior to co-secretion with insulin. A 20-year follow-up study by Fung et al. (42) revealed that increased dietary intake of protein and high-fat dairy products is positively associated with higher plasma C-peptide levels and directly associated with the risk of colorectal cancer. While another study presented by Seidelmann et al. (43) concluded the presence of a U-shaped association between the percentage of energy consumed from carbohydrates and mortality, as they reported that both low-carbohydrate consumption (<40%) and high-carbohydrate consumption (>70%) presented a greater risk of mortality than moderate carbohydrate intake. Even though the role of C-peptide in the regulation of inflammation remains controversial, in our study, we observed a significant correlation between lower carbohydrate intake and higher plasma C-peptide. Multiplex analysis of several inflammatory cytokines further revealed that plasma C-peptide upregulation correlated positively with plasma FGF2, IP-10, IL-6, MDC, and IL-17A levels. Notably, these cytokines have been previously identified to induce the development of insulin resistance and cause the pathogenesis of T2DM (44–48). However, a negative correlation was observed between the C-peptide levels and the expression of the anti-inflammatory cytokine IL-3, a pleiotropic regulator of inflammation (49).

Nevertheless, the present study is limited by certain caveats. In this study, sample collection was achieved randomly and not systemically. Even though such an analysis provides a better approximation of the entire population, several limitations should be considered. For instance, the dietary intake in this study was assessed through self-reported diary logs and not by intervention. Even though adequate training was given to each participant along with a food scale, we could not possibly rule out false reporting. We also have no record of how long each individual would have maintained this dietary lifestyle beyond the 7-day follow-up period. Therefore, the effects of long-term vs. short-term dietary interventions involving carbohydrate intake may not be evaluated. Nevertheless, the most substantial limitation found in this study was the sample size in the HC group. Throughout the investigation, it became clear that a U-shaped effect was observed, indicating that both LC and HC intake reflect unwanted outcomes. This observation falls in line with observations made by other groups (43). However, owing to the small number of participants at the higher end of carbohydrate intake (<70%), it was difficult to reach statistical significance in this group. It is also crucial to note that, although there is a significant correlation between low carbohydrate consumption and IRS risk indicators, this should not be interpreted as a direct impact but rather as a contributory behavioral factor that could enhance the probability of such an outcome if continued uninterrupted over time.

All in all, the presented data highlight, for the first time, the effect of low carbohydrate intake on factors related to IRS in the normal-weight population. We have shown that individuals of a normal weight consuming <45% of their daily energy intake from carbohydrates had trends of dysregulated glucose homeostasis with elevated plasma C-peptide levels and higher anion gap metabolic acidosis. Under the consumption of low carbohydrate intake, we also observed the upregulation of T2DM-related inflammatory markers that were found to significantly correlate with C-peptide levels.

## Data availability statement

The original contributions presented in the study are included in the article/Supplementary material, further inquiries can be directed to the corresponding authors.

## Ethics statement

The studies involving human participants were reviewed and approved by Kuwait Ministry of Health (MOH) Ethics Board (2017/542). The patients/participants provided their written informed consent to participate in this study.

## Author contributions

FA-R conceived the idea, guided the research study, provided material support, procured funds, collected and analyzed data, and wrote the manuscript. SS participated in performing some statistical analysis, writing, and reviewing the manuscript. AA participated in performing experiments and analyzing data. FB, HA, and NA participated in performing experiments and data collection. MM participated in performing statistical analysis and statistical methodology and contributed to writing and reviewing. FA participated in performing some statistical analysis and in writing and reviewing the manuscript. FA-M reviewed and critically commented on the manuscript. RA guided the research study, provided material support, procured funds, wrote, edited, and approved the manuscript for submission. All authors contributed to the article and approved the submitted version.
