# Supplementary material for: Low carbohydrate intake correlates with trends of insulin resistance and metabolic acidosis in healthy lean individuals
Source: Front Public Health. 2023 Mar 16;11:1115333. doi: 10.3389/fpubh.2023.1115333 (PMC10061153; doi:10.3389/fpubh.2023.1115333)
Supplement: Supplementary file 1 [file Image_1.pdf]

Supplementary Material

Low carbohydrate intake associates with trends of insulin resistance and metabolic acidosis in healthy lean individuals

Fatema Al-Reshed[1*], Sardar Sindhu[2], Ashraf Al Madhoun[2,3], Fatemah Bahman[1], Halemah AlSaeed[1], Nadeem Akhter[1], Fawaz Alzaid[4], Fahd Al-Mulla[3], Rasheed Ahmad[1]*

Correspondence: Fatema Alrashed, Ph.D., e-mail: fatema.alrashed@dasmaninstitute.org, Rasheed Ahmad, e-mail: Rasheed.Ahmad@dasmaninstitute.org

1 Supplementary Data

1.1 Supplementary Figures

Supplemental Figure. 1

(A) Illustration diagram for the study design

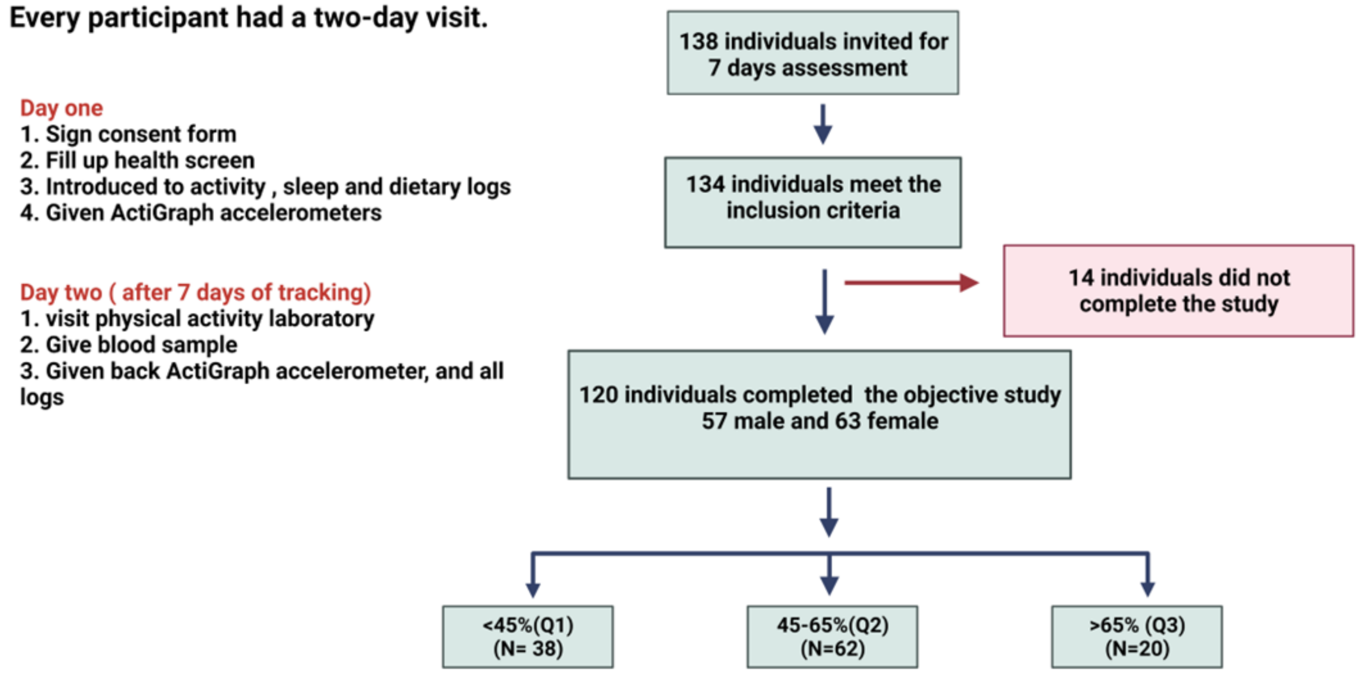

(B) Data adjusted for age sex, total energy intake and physical activity using spearmen correlation

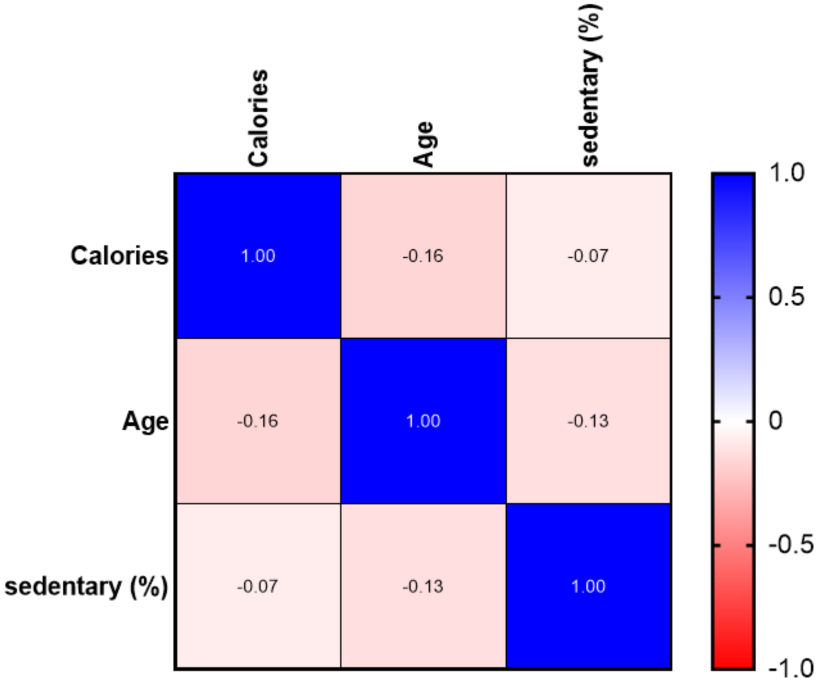

Supplementary Figure 1 Self-reported dietary logs from 120 participants were analyzed and divided three categories according to their carbohydrate intake. Low carbohydrate (LC) (those consuming less than 45% of daily energy percentage), recommended range of carbohydrate (RC) (those consuming less than 45- 65% of daily energy percentage) and high carbohydrate (HC) (those consuming higher than 65% of daily percentage) (A) Flow chart of participant recruitment. (B) Collected data were adjusted for age sex, total energy intake and physical activity using spearmen r correlation**.**
