# Supplementary material for: Low carbohydrate intake correlates with trends of insulin resistance and metabolic acidosis in healthy lean individuals
Source: Front Public Health. 2023 Mar 16;11:1115333. doi: 10.3389/fpubh.2023.1115333 (PMC10061153; doi:10.3389/fpubh.2023.1115333)
Supplement: Supplementary file 2 [file Data_Sheet_1.PDF]

Supplementary Table 1: Physical characteristics of study participants; male vs female

Parameter	Males (N= 57)	Females (N=63)	p-value
Age (years)	31.9 ± 5.2	32.5 ± 5.0	0.5262
Weight (kg)	69.4 ± 12.6	60.5 ± 10.0	<0.0001
Height (cm)	176.3 ± 9.3	160.3 ± 8.2	<0.0001
BMI (kg/m^2)	22.7 ± 2.4	23.1 ± 2.7	0.4104
Waist circumference(inch)	31.7 ± 3.7	29.9 ± 3.8	0.0148
Hip circumference (inch)	40.3 ± 9.8	40.1 ± 4.1	0.8589
Fat weight (kg)	21.1 ± 11.5	25.0 ± 9.5	0.0246
Lean weight (kg)	57.9 ± 9.4	43.2 ± 9.1	<0.0001
Body fat (%)	22.2 ± 9.3	27.7 ± 5.7	<0.0001
BP/ systolic (mmHg)	111.9 ± 11.4	108 ± 11	0.0596
BP/diastolic (mmHg)	68.9± 8.7	70.2 ± 8.2	0.4014
Heart rate (HR)	71.1 ± 8.4	72.0 ± 7.1	0.5197
Waist to hip ratio	0.8 ± 0.11	0.76 ± 0.7	0.0143
Fasting blood glucose (mmol/l)	4.9 ± 0.54	4.8 ± 0.73	0.4647
Triglycerides (mmol/l)	0.83 ± 0.43	0.92 ± 0.36	0.1847
Total cholesterol (mmol/l)	4.38 ± 0.68	4.56 ± 0.82	0.1525
HDL cholesterol (mmol/l)	1.51 ± 0.36	1.52 ± 0.36	0.8098
Insulin Conc. (mu/l)	3.85 ± 2.15	4.5 ± 2.0	0.0616
HOMA-IR	0.84 ± 0.45	0.98 ± 0.43	0.1051
C-Peptide	1.27 ± 0.33	1.25 ± 0.33	0.7577
Total Calories/day (kcal)	2181 ± 588	1972 ± 540	0.0449
Carbs (g)	272.4 ±76.5	256.8 ±1 11.9	0.3750
Fat (g)	68.5 ± 31	55 ± 25.7	0.0324
Protein (g)	99.3 ± 42.8	81.9 ± 30.3	0.0348
Chol (mg)	226.6 ± 148.2	155.3 ± 111.2	0.0139
Sodium (mg)	1375.8 ± 397.7	1450.4 ± 523.5	0.4494
Sugars (g)	106.2 ± 91.5	65.7 ± 39.1	0.0120
Fiber (g)	25.3 ± 17.5	19 ± 14.9	0.0790

Supplementary Table 2: Normal ranges

Parameter	Normal range
Fasting blood glucose (mmol/l)	3.9 - 6.4
Triglycerides (mmol/l)	0.37 - 2.3
Total cholesterol (mmol/l)	0 - 5.2
HDL cholesterol (mmol/l)	0.83 - 1.83
Insulin Conc. (mu/l)	5 - 15
HOMA-IR	≤1
C-Peptide (ng/ml)	0.78 – 1.89

Supplementary Table 3: Charactesuristics of study participants

Parameter	All participants (n=120)	Males (n= 57)	Females (n=63)	p-value male to Female
Age (years)	32.2 ± 5.7	31.9 ± 5.2	32.5 ± 5.0	0.526
Weight (kg)	65.0 ± 12.1	69.4 ± 12.6	60.5 ± 10.0	<0.0001
Height (cm)	168.3 ± 11.8	176.3 ± 9.3	160.3 ± 8.2	<0.0001
BMI (kg/m^2)	22.7 ± 2.4	22.7 ± 2.4	23.1 ± 2.7	0.4104
Waist circumference(inch)	29.4 ± 3.6	31.7 ± 3.7	29.9 ± 3.8	0.0148
Hip circumference (inch)	40.3 ± 9.8	40.3 ± 9.8	40.1 ± 4.1	0.8589
Fat weight (kg)	23.3 ± 11.9	21.1 ± 11.5	25.0 ± 9.5	0.0246
Lean weight (kg)	45.7 ± 9.7	57.9 ± 9.4	43.2 ± 9.1	<0.0001
Body fat (%)	25.7 ± 9.3	22.2 ± 9.3	27.7 ± 5.7	<0.0001
BP/ systolic (mmHg)	109.9 ± 11.3	111.9 ± 11.4	108 ± 11	0.0596
BP/diastolic (mmHg)	67.15± 9.8	68.9± 8.7	70.2 ± 8.2	0.4014
Heart rate (HR)	71.4 ± 10.9	71.1 ± 8.4	72.0 ± 7.1	0.5197
Waist to hip ratio	32.2 ± 5.7	0.8 ± 0.11	0.76 ± 0.7	0.0143
Fasting blood glucose (mmol/l)	4.9 ± 0.64	4.9 ± 0.54	4.8 ± 0.73	0.027
Triglycerides (mmol/l)	0.87 ± 0.38	0.83 ± 0.43	0.92 ± 0.36	0.184
Total cholesterol (mmol/l)	4.6 ± 0.8	4.38 ± 0.68	4.56 ± 0.82	0.152
HDL cholesterol (mmol/l)	1.49 ± 0.34	1.51 ± 0.36	1.52 ± 0.36	0.809
Insulin Conc. (mu/l)	3.7 ± 2.11	3.85 ± 2.15	4.5 ± 2.0	0.061
HOMA-IR	0.82 ± 0.48	0.84 ± 0.45	0.98 ± 0.43	0.081
C-Peptide	1.26 ± 0.37	1.27 ± 0.33	1.25 ± 0.33	0.758
Total Calories/day (kcal)	2143.8 ± 571.9	2181 ± 588	1972 ± 540	0.048
Carbs (g)	264.6 ± 95.8	272.4 ±76.5	256.8 ±1 11.9	0.260
Fat (g)	49.5 ± 12.5	68.5 ± 31	55 ± 25.7	0.032
Protein (g)	56.7 ± 28.0	99.3 ± 42.8	81.9 ± 30.3	0.035
Chol (mg)	19.2 ± 10.3	226.6 ± 148.2	155.3 ± 111.2	0.021
Sodium (mg)	91.2 ± 48.8	1375.8 ± 397.7	1450.4 ± 523.5	0.739
Sugars (g)	17.8 ± 10.1	106.2 ± 91.5	65.7 ± 39.1	0.012
Fiber (g)	197.2 ± 138.1	25.3 ± 17.5	19 ± 14.9	0.079
